# Associations of appetitive traits with growth velocities from infancy to childhood

**DOI:** 10.1038/s41598-023-42899-0

**Published:** 2023-09-25

**Authors:** Duaa Ibrahim Olwi, Felix R. Day, Tuck Seng Cheng, Laurentya Olga, Clive J. Petry, Ieuan A. Hughes, Andrea D. Smith, Ken K. Ong

**Affiliations:** 1 MRC Epidemiology Unit, Wellcome Trust-MRC Institute of Metabolic Science, University of Cambridge, School of Clinical Medicine, Box 285, Cambridge, CB2 0QQ UK; 2 King Abdullah International Medical Research Center, Jeddah, Saudi Arabia; 3 King Saud Bin Abdulaziz University for Health Sciences, Jeddah, Saudi Arabia; 4 Department of Paediatrics, University of Cambridge, Cambridge, UK

**Keywords:** Epidemiology, Paediatric research

## Abstract

Several studies have reported associations between appetitive traits and weight gain during infancy or childhood, but none have directly compared these associations across both age periods. Here, we tested the associations between appetitive traits and growth velocities from birth to childhood. Appetitive trait data were collected using the Children’s Eating Behaviour Questionnaire (CEBQ) in 149 children from the Cambridge Baby Growth Study at age 9–17 years. These participants also provided anthropometric measurements during infancy (birth, 3, 12, 18, and 24 months) and childhood (5 to 11 years). Standardized growth velocities (in weight, length/height, BMI, and body fat percentage) for 0–3 months, 3–24 months, and 24 months to childhood were estimated using individual linear-spline models. Associations between each of the eight CEBQ traits and each growth velocity were tested in separate multilevel linear regression models, adjusted for sex, age at CEBQ completion, and the corresponding birth measurement (weight, length, BMI, or body fat percentage). The three food-approach traits (*food responsiveness*, *enjoyment of food* and *emotional overeating*) were positively associated with infancy and childhood growth velocities in weight, BMI, and body fat percentage. By contrast, only one of the food-avoidant traits, *satiety responsiveness*, was negatively associated with all growth velocities. Significant associations were mostly of similar magnitude across all age periods. These findings reveal a broadly consistent relationship between appetitive traits with gains in weight and adiposity throughout infancy and childhood. Future interventions and strategies to prevent obesity may benefit from measuring appetitive traits in infants and children and targeting these as part of their programs.

## Introduction

Childhood obesity is associated with a multitude of negative health implications. Weight status in childhood tracks into adolescence and eventually adulthood. Children living with obesity are at greater risk of continuing to do so later in life. This suggests that preventive efforts are best targeted at the early years to maximise impact^1^. Appetitive traits are key behaviours linked with obesity, which comprise a range of food-approach and food-avoidant eating behaviours^2^. Characterising which appetitive traits are associated with greater velocity of weight gain across key developmental phases in infancy and childhood could help in identifying potential behavioural targets for interventions to prevent childhood obesity.

Appetitive traits are innate tendencies that are observable early in infancy and show continuity and stability throughout childhood^3–5^. A number of psychometric instruments have been developed to assess these traits in children^6, 7^. One of the most comprehensive psychometric measures is the Children’s Eating Behaviour Questionnaire (CEBQ)^2^. The CEBQ is a validated parent-reported instrument composed of 35 items which are used to calculate four ‘food-approach’ appetitive traits (*food responsiveness*, *enjoyment of food*, *emotional overeating*, and *desire to drink*) and four ‘food-avoidant’ appetitive traits (*satiety responsiveness*, *slowness in eating*, *emotional undereating*, and *food fussiness*). Although the food-approach traits tend to increase with age and the food-avoidant traits tend to decrease with age^2^, these changes are relatively small and individual children tend to hold their relative ranking in regards to appetite traits compared to their peers^3, 5^. A growing body of evidence underscores the substantial heritability of appetitive traits from birth onwards. For example, during the exclusively milk fed period of life in infants, genetic influences already account for approximately 53–84% of inter-individual variability in four appetitive traits^8^. This substantial proportion of genetic influence remain relatively stable with heritability estimates ranging between 69 and 90% for all CEBQ traits except *emotional undereating* in school aged-children^9^. However, as children mature and gain greater autonomy in their dietary decisions, their ability to express their genetic predisposition for heightened appetite may become more pronounced^9^. In an obesogenic and food permissive environment, this predisposes to increased dietary energy intake and, consequently, risk for obesity^10^.

Accumulating evidence supports the relevance of CEBQ traits to obesity risk in childhood. Longitudinal studies report that a more avid appetite is prospectively associated with faster growth in childhood (aged 6 to 10 years), indexed by higher body mass index (BMI)^11–14^, fat mass index (FMI), and fat-free mass index (FFMI)^11^. On the other hand, food avoidant appetitive traits are associated with subsequent lower childhood growth in children aged 4 to 10 years (BMI^15–17^, FMI, and FFMI^11, 16^). Similar findings have been reported regarding appetitive traits measured during infancy using the Baby Eating Behaviour Questionnaire (BEBQ) at 3 months and their subsequent association with infant weights and BMI up to 15 months of age^18–20^. Conversely, adiposity could also influence appetitive traits, where children of higher adiposity may develop increasingly avid appetite^21^. It is suggested that appetite plays a more important role in adiposity (than does adiposity in appetite development) particularly in early infancy, while the relationship becomes more complex during childhood^22^. To date, however, most studies, both cross-sectional and longitudinal, have focused on BMI as an index of adiposity^21^, which fails to differentiate between lean and fat mass^23^.

Recent studies have reported a relationship between appetitive traits and dietary intakes, with possible consequences for growth. In the PANIC study (n = 406 children; aged 6–8 years), *enjoyment of food* and *food responsiveness* were positively associated with intakes of nutrient-dense and protein-rich foods, assessed by 4-day food records, whereas *satiety responsiveness* and *food fussiness* were negatively associated with those food intakes^24^. In the Generation XXI study (n = 3879 children; 7–10 years), higher *enjoyment of food* and lower *satiety responsive* and *food fussiness* were associated with a higher diet quality, as evaluated by the Healthy Eating Index^25^. However, there are no data on the links between appetitive traits and growth from birth with consideration of both infancy and childhood growth trajectories.

Therefore, the present study aimed to assess the association between appetitive traits, as assessed by the CEBQ in children aged 9–17 years (mean age 12.5 ± 1.4 years), and growth patterns during both infancy and childhood (in weight, length/height, BMI, and body fat percentage). We hypothesized that appetitive traits would show similar associations with growth in both infancy and early childhood.

## Methods

### Study population

**Figure 1:**
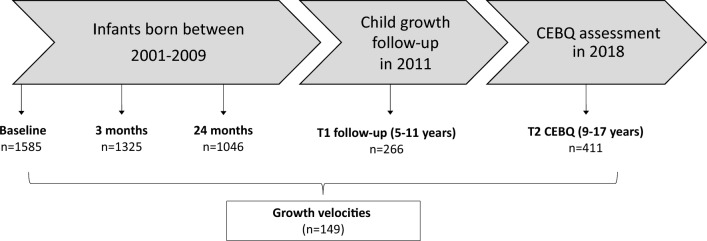
Flow diagram showing numbers of participating infants and children. CEBQ, Child Eating Behaviour Questionnaire; T, time.

The Cambridge Baby Growth Study (CBGS) is a birth cohort of infants born between 2001 and 2009 to mothers recruited from the Rosie Maternity Hospital, Cambridge, England. Mothers aged 16 years and older and able to give consent were eligible to participate, where all experiments were performed in accordance with relevant guidelines and regulations. Routine data on infant sex, ethnicity, and gestational age were collected at birth, and the mode of breastfeeding was reported by parents at the 3-month clinic visit. Self-reported maternal educational attainment was categorized into high (university degree or higher), intermediate (vocational education or diploma), or low (high school education or less). Anthropometry of the child was assessed repeatedly during infancy (at birth, 3, 12, 18 and 24 months) and at one childhood clinic visit (at 5 to 11 years). The CEBQ was administered by post in 2018 to assess the children’s present appetitive traits. At the time, the children were aged between 9 and 17 years old, with a mean age of 12.5 ± 1.4 years. The sampling frame for this analysis was the 149 children who had growth velocities calculated at both infancy and childhood, and completed the CEBQ (Fig. 1).

### Appetitive traits

The CEBQ is a validated parent-reported questionnaire with a robust factor structure, internal reliability, and test–retest reliability^2^. It was administered to children aged 9 to 17, with a mean age of 12.5 ± 1.4 years. The CEBQ comprises eight subscales (traits) ascertained by 35 items answered using a 5-point Likert scale, ranging from 1 (never) to 5 (always). *Food responsiveness* (5 items) and *enjoyment of food* (4 items) indicate the child’s likelihood and enthusiasm of eating in response to food cues. *Desire to drink* (3 items) indicates the child’s preference to consume beverages, typically sweetened drinks. *Emotional overeating* (4 items) and *emotional undereating* (4 items) indicate eating tendencies in response to intense emotions. *Satiety responsiveness* (5 items) indicates the child’s perception of feelings of fullness. *Slowness in eating* (4 items) and *food fussiness* (6 items) indicate speed of eating or lack of interest in food in response to its visual presentation, texture or taste, respectively^2, 3^. A mean score was calculated for each subscale, with higher scores indicative of greater expression of that appetitive trait.

### Growth parameters

Anthropometry was performed by trained research nurses at (mean ± SD) ‘3 months’ (3.2 ± 0.3 months), ‘12 months’ (12.5 ± 0.5 months), ‘18 months’ (18.5 ± 0.5 months), ‘24 months (24.2 ± 0.4 months), and ‘childhood’ (9.5 ± 1.1 years; range 5 to 11 years). Weight was measured to the nearest 1 g during infancy and 0.1 kg during childhood. Length/height was measured to the nearest 0.1 cm. Skinfold thickness was measured in triplicate at four body sites (triceps, subscapular, flank, and quadriceps). Total body fat mass was estimated during infancy using validated equations (fat mass in kilograms = 2.167 + 0.512 * weight in kilograms + 0.041 * triceps skinfold (SF) in millimeters + 0.008 * subscapular SF in millimeters + 0.011 * flank (suprailiac) SF in millimeters + 0.002 * age at visit in days – 0.074 * length in centimeters – 0.037 * sex (1 = male; 0 = female))^26^. At the childhood visit, whole-body dual-energy x-ray absorptiometry (DEXA) was used to assess total body fat mass^27^.

Growth parameters were weight, length/height, BMI (calculated by dividing weight in kilograms by height in meters squared), and body fat percentage (calculated by dividing total fat mass in kilograms by body weight in kilograms, and then multiplying by 100). Weight, length/height, and BMI values were converted to age- and sex-standardized z-scores by comparison to the British 1990 growth reference^28, 29^, using the Stata zanthro package. For body fat percentage, at each age, internally derived z-scores were calculated as the standardized residuals of linear regression models that included age and sex as covariates. Measurements at birth and 3 months of age were additionally adjusted for gestational age.

### Growth velocities

Linear-spline multilevel models at the individual level (also known as piecewise linear models) were performed to estimate growth velocities (expressed as change in z-score per month) during each growth interval for weight, length/height, BMI, and body fat percentage. Knot points were chosen at 3 months and 24 months based on visual inspection of the data, as previously described^27^, giving three growth intervals: 0–3 months, 3–24 months, and 24 months to childhood.

### Statistical analysis

Univariate distributions for all variables were presented as means and standard deviations for continuous variables and as frequency and percentages for categorical variables. To test the associations of growth velocities with each CEBQ trait, multivariable linear regression models were performed. The models included the combined growth intervals for weight, length/height, BMI, or body fat percentage, and as covariates: sex, age at CEBQ completion, and the corresponding standardized birth measurements (weight, length, BMI, or body fat percentage). Given that there might be age range-specific associations between the eating behaviour and growth, we wanted to assess the heterogeneity across the age groups. To do this, we used a post-estimation Wald test between the growth velocities estimated in each of 0–3 months, 3–24 months, and 24 months to childhood.

All tests were two sided and performed using Stata version 16 (StataCorp, College Station, TX) or R version 3.6.2 (The R Foundation for Statistical Computing, Vienna, Austria). *P* values < 0.05 were considered statistically significant.

### Ethics declarations

Written informed consent was provided by mothers and the study was approved by the local Cambridge research ethics committee.

## Results

### Sample characteristics

Descriptive characteristics of the 149 children with data on CEBQ and anthropometric measurements during both infancy and childhood are summarized in Table 1, compared to excluded children (n = 1436). The included and excluded samples were similar for all characteristics, except for maternal education, which was lower in the excluded sample.

**Table 1:** Characteristics of the included analysis sample and excluded CBGS children.

	Mean (SD)	
	Analysis sample	Excluded children
Characteristic	(n = 149)	(n = 1436)
Boys, no. (%)	63 (42.3)	750 (52.5)
Ethnicity, no. (%)		
European	114 (97.4)	912 (95.9)
Asian	3 (2.6)	18 (1.9)
Black	–	11 (1.2)
Other	–	10 (1.0)
Missing/not specified	32	485
Maternal educational level, no. (%)		
High	61 (41.0)	414 (28.8)
Intermediate	16 (10.7)	267 (18.6)
Low	72 (48.3)	755 (52.6)
Maternal age at birth, years	33.7 (4.2)	33.5 (4.3)
Gestational age, weeks	40.1 (1.4)	39.8 (1.6)
Exclusive breastfeeding, weeks	5.9 (4.3)	4.9 (4.3)
Age 0 months		
Weight, kg	3.5 (0.4)	3.5 (0.5)
Length/height, cm	51.4 (2.3)	51.4 (2.6)
Body fat percentage	13.9 (4.4)	13.3 (5.5)
Age 3 months		
Weight, kg	6.1 (0.8)	6.2 (0.8)
Length/height, cm	61.2 (2.7)	61.1 (2.6)
Body fat percentage	23.5 (3.7)	23.8 (3.5)
Age 24 months		
Weight, kg	12.6 (1.3)	12.6 (1.5)
Length/height, cm	87.8 (3.3)	87.8 (3.5)
Body fat percentage	32.6 (1.9)	32.7 (2.0)
Childhood (5 to 11 years)		
Age (years)	9.5 (1.1)	9.5 (1.1)
Weight, kg	32.6 (7.0)	32.9 (6.9)
Length/height, cm	139.2 (8.9)	138.8 (9.0)
Body fat percentage	22.4 (7.9)	23.5 (8.4)
CEBQ traits^a^		
Age (years)	12.5 (1.5)	13.0 (0.9)
Food responsiveness	2.4 (0.8)	2.5 (0.9)
Enjoyment of food	4.0 (0.7)	3.9 (0.7)
Emotional overeating	1.9 (0.7)	2.1 (0.7)
Desire to drink	2.0 (0.7)	2.1 (0.8)
Satiety responsiveness	2.5 (0.7)	2.5 (0.7)
Slowness in eating	2.4 (0.8)	2.4 (0.8)
Emotional undereating	2.5 (0.7)	2.6 (0.8)
Food fussiness	2.5 (0.9)	2.7 (1.0)

CEBQ: Child Eating Behaviour Questionnaire; SD: standard deviation.

^a^CEBQ subscale scores in 411 children.

Correlations between the CEBQ traits among all children who collected such data (n = 411) are shown in Supplementary Table 1. The food-approach appetitive traits (*food responsiveness*, *enjoyment of food*, *emotional overeating*, and *desire to drink*) showed variable weak to moderate positive intercorrelations (r = 0.05 to 0.56) as did the food-avoidant appetitive traits (*satiety responsiveness*, *slowness in eating*, *emotional undereating*, and *food fussiness*) (r = 0.09 to 0.49). The correlations between the food-approach and food-avoidant appetitive traits were mostly negative, except for positive correlations between *emotional overeating* and *emotional undereating* (r = 0.36) and between *desire to drink* and *emotional undereating* (r = 0.17).

### Associations between appetitive traits and growth velocities

#### Food-approach appetitive traits

**Figure 2:**
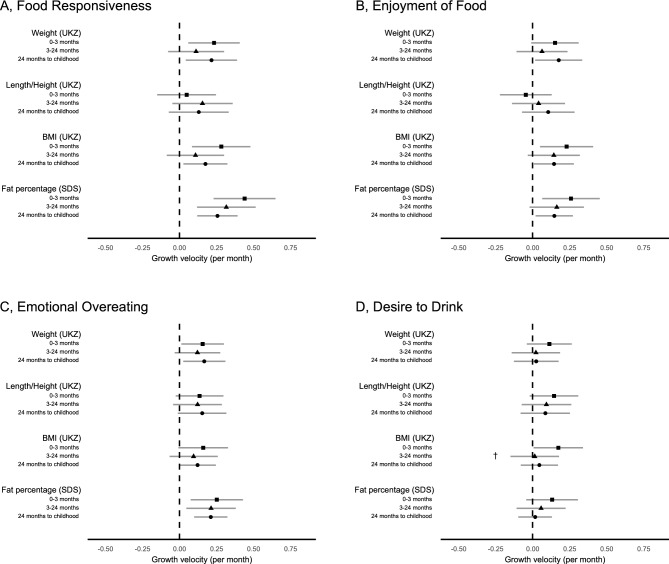
Associations between the food-approach appetitive traits (**A**, food responsiveness; **B**, enjoyment of food; **C**, emotional overeating; **D**, desire to drink) and individual-level growth velocities (UK z-score change/month for 1-unit increase in Likert scale) for weight, length/height, BMI, and (standard deviations of) body fat percentage. All models were adjusted for sex, age at the questionnaire completion, and birth measurements (weight, length/height, BMI, or body fat percentage). Error bars display the 95% confidence intervals of each estimate. ^†^Associations with growth velocities at 0–3 months, 3–24 months, and 24 months to childhood (5 to 11 years) were statistically heterogenous (Wald test *P* < 0*.*05). UKZ, UK z-score; SDS, standard deviation score.

Figure 2 and Supplementary Table 2 show the associations between parent-perceived food-approach appetitive traits at 9–17 years old and growth velocities at different intervals from birth to childhood. *Food responsiveness* was positively associated with growth velocities in weight and BMI at 0–3 months (β = 0.23 UK z-score change/month for 1-unit increase in Likert scale, *P* = 0.009 and β = 0.28 UK z-score change/month, *P* = 0.005, respectively) and 24 months to childhood (β = 0.22 UK z-score change/month, *P* = 0.015 and β = 0.18 UK z-score change/month, *P* = 0.021, respectively), and in body fat percentage at all growth intervals. *Enjoyment of food* was positively associated with growth velocities in BMI and body fat percentage at 0–3 months and 24 months to childhood, and in weight at 24 months to childhood. *Emotional overeating* was positively associated with velocities in weight at 0–3 months and 24 months to childhood, and in body fat percentage at all ages. These food-approach appetitive traits showed no evidence of heterogeneity across the different growth periods (0–3 months, 3–24 months, and 24 months to childhood). *Desire to drink* showed no association with any growth velocity except with BMI only at 0–3 months.

#### Food-avoidant appetitive traits

**Figure 3:**
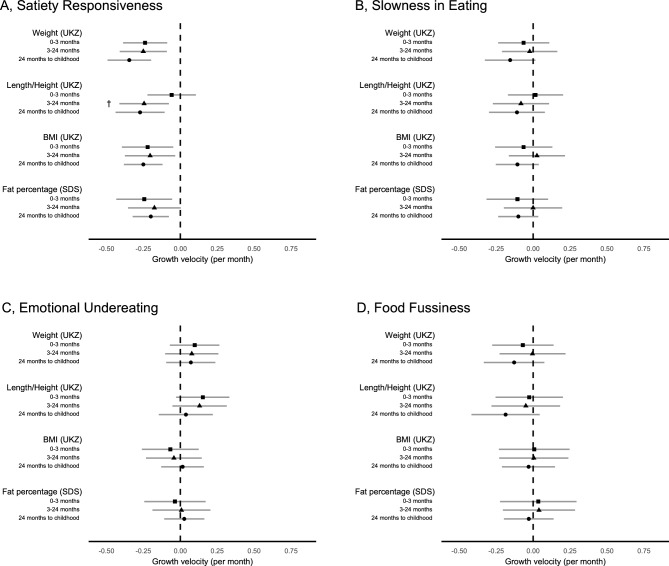
Associations between the food-avoidant appetitive traits (**A**, satiety responsiveness; **B**, slowness in eating; **C**, emotional undereating; **D**, food fussiness) and individual-level growth velocities (UK z-score change/month for 1-unit increase in Likert scale) for weight, length/height, BMI, and (standard deviations of) body fat percentage. All models were adjusted for sex, age at the questionnaire completion, and birth measurements (weight, length/height, BMI, or body fat percentage). Error bars display the 95% confidence intervals of each estimate. ^†^Associations with growth velocities at 0–3 months, 3–24 months, and 24 months to childhood (5 to 11 years) were statistically heterogenous (Wald test *P* < 0*.*05). UKZ, UK z-score; SDS, standard deviation score.

Figure 3 and Supplementary Table 3 show the associations between parent-perceived food-avoidant appetitive traits at 9–17 years old and growth velocities at different intervals from birth to childhood. Of the food-avoidant appetitive traits, only *satiety responsiveness* was negatively associated with velocities in weight (0–3 months: β = − 0.24 UK z-score change/month for 1-unit increase in Likert scale, *P* = 0.002; 3–24 months: β = − 0.25 UK z-score change/month, *P* = 0.002; 24 months to childhood: β = − 0.34 UK z-score change/month, *P* = 8 × 10^–6^) and BMI (0–3 months: β = − 0.22 UK z-score change/month, *P* = 0.013; 3–24 months: β = − 0.2 UK z-score change/month, *P* = 0.019; 24 months to childhood: β = − 0.25 UK z-score change/month, *P* = 2 × 10^–4^) at all ages, and in body fat percentage at 0–3 months and 24 months to childhood. *Satiety responsiveness* was also negatively associated with height velocity at 3–24 months and 24 months to childhood, but not at 0–3 months (Wald test *P*-heterogeneity = 0.047).

## Discussion

In this study, we modelled associations between parent-reported food-approach and food-avoidant appetitive traits with objectively measured growth velocities at different time points across child development. Consistent with previous research, we showed that the food-approach appetitive traits (i.e. *food responsiveness, enjoyment of food,* and *emotional overeating*) were positively associated with gains in weight, BMI, and body fat percentage at most ages. These relationships were not seen for length or child height. Furthermore, the food-avoidant appetitive traits (i.e. *satiety responsiveness)* showed negative associations with most growth velocities. The associations with growth velocities were predominantly consistent in effect size across infancy and childhood.

Our findings are consistent with previous reports, which showed that greater expression of *food responsiveness* and *lower satiety responsiveness* were associated with higher weight gain in infancy^18, 19^ and higher BMI in childhood^12, 14, 15, 20^. Although appetite and satiety are partially controlled by neurologically dissociable systems, there is a considerable interplay between the two^11^. This is supported by the negative correlations between some food-approach and food-avoidant traits in our study and also in previous reports^2, 30, 31^. The positive association between *enjoyment of food* with gains in BMI seen in our study is consistent with findings of an earlier systematic review that investigated prospective associations between *enjoyment of food* with BMI z-score and percentile^21^. This aligns with previous research showing that appetitive traits are considered to be innate predispositions, which in obesogenic environments elevates the risk for rapid weight gain^21, 22^.

Consistent with some earlier longitudinal studies^11, 13, 14^, *emotional overeating* was positively associated with BMI gains, however *emotional undereating* showed no association with any growth velocity. Despite their different patterns of association with obesity, *emotional overeating* and *emotional undereating* were positively correlated, which aligns with previous data^2, 32, 33^. Both traits indicate eating behaviour reactions to emotions, usually negative emotions such as stress or sadness^32, 34^.

To our knowledge, the associations between the CEBQ traits and body fat percentage are rarely explored. We found appetitive traits had stronger associations with body fat percentage compared to other anthropometric measures. We observed associations of *food responsiveness*, *enjoyment of food*, and *emotional overeating* (all food-approach traits) and *satiety responsiveness* (a food-avoidant trait) with changes in body fat percentage. This might suggest that prior studies employing BMI instead of adiposity measures could potentially have underestimated the associations with appetitive traits. Furthermore, these findings could indicate a distinct connection between appetitive traits and adiposity, as opposed to overall body size and growth. There is indirect support for this hypothesis from other studies that reported links between *food responsiveness* and liking of non-core foods, which tend to be of higher caloric density^35, 36^, and between *emotional eating* with intakes of palatable energy-dense foods^37^ and sugar-sweetened drinks^38^. The strong association between appetitive traits and body fat percentage underscores the potential of using eating behaviour as a tool to differentiate between lean mass and fat mass in the context of childhood obesity.

A key insight in the present study is associations between appetitive traits and growth from infancy to childhood. Although previous longitudinal studies showed similar findings on the association between eating behaviour and growth during infancy or childhood^11–20^, none has investigated relationships spanning both age periods. Our findings suggest a broadly consistent link between appetitive traits and various dimensions of growth across both infancy and childhood. This highlights the potential of using appetitive traits for predicting childhood obesity.

We found that *slowness in eating* and *food fussiness* were not associated with growth velocity. This differs from the systematic review and meta-analysis by Kininmonth et al., which reported that *slowness in eating* was consistently negatively associated with adiposity whereas *food fussiness* showed null associations^21^. It may be relevant to note that, in general, *slowness in eating* declines with age in longitudinal studies, as children become more proficient at eating^3^, and we administered the CEBQ at relatively older ages than other studies. The food-avoidant trait *food fussiness* indicates the innate tendency to be selective about the foods a child is willing to try, often focusing on attributes such as texture or presentation^39^. It tends to be most pronounced during late infancy and then reduces with age as a result of repeated exposure to foods^39^.

We acknowledge several limitations in our study. First, we administered the CEBQ at only one time point that spanned a wide range of ages, and after the growth measurements. This is challenging as appetitive traits are to some extent dynamic and the age at CEBQ measurement influences the interpretation of the observed relationships. Furthermore, the CEBQ has not been validated in older children (> 14 years of age). However, there is evidence that childhood appetitive traits track and show stability from infancy to childhood^3–5^. Moreover, as children mature and attain increased autonomy in their food choices, their ability to express genetic predisposition for heightened appetite might become more evident^10^. Secondly, the single measurement of appetitive traits limited our ability to assess their likely causal direction with growth. One key area of research in this field relates to better understating the bidirectionality between eating behaviour and adiposity^21^. However, due to the single CEBQ measurement, the results presented in this study are not able to disentangle directionality of the observed consistent associations. Furthermore, most children in this study were of White European origin and were recruited from a single center, which may reduce generalizability. That being said, the growth parameters of the CBGS sample were comparable to the UK population-based growth references^29^. A key strength of our study was the comprehensive range of anthropometric indicators included in the analyses, including four skinfolds and DEXA scans. This allows for a more objective understanding of the link between eating behaviours and adiposity development. These growth data have been shown to have low relative intra-observer technical errors of measurements^40^. Lastly, the CEBQ was parent-reported, and parental perceptions and social desirability bias need to be taken into account.

## Conclusions

This study shows positive and negative associations of food-approach and food-avoidant appetitive traits at 9–17 years with growth velocities from birth to childhood, respectively. Given the potentially stable appetitive traits suggested by several studies, this study suggests the relevance of appetitive traits to growth velocities of adiposity-related traits, demonstrating broadly consistent relationships from infancy to childhood. Future research is necessary to determine the potential of mitigating the effects of appetitive traits in reducing risk for childhood obesity.

### Supplementary Information

Supplementary Tables.

## Data Availability

The datasets used and/or analysed during the current study are available from the corresponding author on reasonable request.

## Abbreviations

BEBQ: Baby Eating Behaviour Questionnaire
BMI: Body mass index
CBGS: Cambridge Baby Growth Study
CEBQ: Children’s Eating Behaviour Questionnaire
DEXA: Dual-energy x-ray absorptiometry
FFMI: Fat-free mass index
FMI: Fat mass index
SF: Skinfold
