# Supplementary material for: Associations of appetitive traits with growth velocities from infancy to childhood
Source: Sci Rep. 2023 Sep 25;13:16056. doi: 10.1038/s41598-023-42899-0 (PMC10520028; doi:10.1038/s41598-023-42899-0)
Supplement: Supplementary file 1 — Supplementary Tables. [file 41598_2023_42899_MOESM1_ESM.docx]

# **Supplementary content**

**Supplementary Table 1.** Correlations between the CEBQ traits

**Supplementary Table 2.** Associations between the food-approach appetitive traits and growth velocities

**Supplementary Table 3.** Associations between the food-avoidant appetitive traits and growth velocities

**Supplementary Table 1. Correlations between the CEBQ traits in 411 children**

| CEBQ traits | Food responsiveness | Enjoyment of food | Emotional overeating | Desire to drink | Satiety responsiveness | Slowness in eating | Emotional undereating | Food fussiness |
| --- | --- | --- | --- | --- | --- | --- | --- | --- |
| Food responsiveness | 1 [reference] |  |  |  |  |  |  |  |
| Enjoyment of food | 0.40^a^ | 1 [reference] |  |  |  |  |  |  |
| Emotional overeating | 0.56^a^ | 0.09 | 1 [reference] |  |  |  |  |  |
| Desire to drink | 0.31^a^ | 0.05 | 0.23^a^ | 1 [reference] |  |  |  |  |
| Satiety responsiveness | -0.35^a^ | -0.66^a^ | -0.1 | 0.03 | 1 [reference] |  |  |  |
| Slowness in eating | -0.19^a^ | -0.39^a^ | -0.05 | -0.08 | 0.49^a^ | 1 [reference] |  |  |
| Emotional undereating | 0.08 | -0.17^a^ | 0.36^a^ | 0.17^a^ | 0.26^a^ | 0.17^a^ | 1 [reference] |  |
| Food fussiness | 0.08 | -0.54^a^ | 0.05 | 0.05 | 0.36^a^ | 0.19^a^ | 0.09 | 1 [reference] |

^a^ *P* < 0.001

**Supplementary Table 2. Associations between the food-approach appetitive traits and growth velocities^a^ in 149 children**

| Food-approach appetitive traits | Growth periods | Change in weight z-score | |  | Change in length/height  z-score | |  | Change in BMI z-score | |  | Change in body fat percentage z-score | |
| --- | --- | --- | --- | --- | --- | --- | --- | --- | --- | --- | --- | --- |
|  |  | β^b^ (95% CI) | *P* value |  | β^b^ (95% CI) | *P* value |  | β^b^ (95% CI) | *P* value |  | β^b^ (95% CI) | *P* value |
| Food responsiveness | 0-3 months | 0.23 (0.06, 0.41) | 0.009 |  | 0.05 (-0.15, 0.25) | 0.632 |  | 0.28 (0.08, 0.48) | 0.005 |  | 0.44 (0.23, 0.65) | 5*10^-5^ |
|  | 3-24 months | 0.11 (-0.08, 0.3) | 0.242 |  | 0.16 (-0.05, 0.36) | 0.133 |  | 0.11 (-0.09, 0.3) | 0.272 |  | 0.32 (0.12, 0.51) | 0.002 |
|  | 24 months to childhood | 0.22 (0.04, 0.39) | 0.015 |  | 0.13 (-0.07, 0.33) | 0.204 |  | 0.18 (0.03, 0.32) | 0.021 |  | 0.26 (0.12, 0.39) | 3*10^-4^ |
|  |  |  |  |  |  |  |  |  |  |  |  |  |
| Enjoyment of food | 0-3 months | 0.15 (-0.01, 0.31) | 0.064 |  | -0.05 (-0.22, 0.13) | 0.605 |  | 0.23 (0.05, 0.41) | 0.013 |  | 0.26 (0.07, 0.45) | 0.009 |
|  | 3-24 months | 0.06 (-0.11, 0.24) | 0.468 |  | 0.04 (-0.14, 0.22) | 0.66 |  | 0.14 (-0.03, 0.32) | 0.111 |  | 0.16 (-0.02, 0.35) | 0.081 |
|  | 24 months to childhood | 0.18 (0.02, 0.33) | 0.03 |  | 0.11 (-0.07, 0.28) | 0.241 |  | 0.14 (0.01, 0.28) | 0.037 |  | 0.15 (0.02, 0.27) | 0.024 |
|  |  |  |  |  |  |  |  |  |  |  |  |  |
| Emotional overeating | 0-3 months | 0.16 (0.01, 0.3) | 0.034 |  | 0.13 (-0.03, 0.3) | 0.100 |  | 0.16 (-0.01, 0.33) | 0.061 |  | 0.25 (0.08, 0.43) | 0.005 |
|  | 3-24 months | 0.12 (-0.03, 0.28) | 0.123 |  | 0.12 (-0.04, 0.29) | 0.145 |  | 0.1 (-0.07, 0.26) | 0.248 |  | 0.21 (0.05, 0.38) | 0.013 |
|  | 24 months to childhood | 0.17 (0.03, 0.31) | 0.021 |  | 0.15 (-0.01, 0.32) | 0.067 |  | 0.12 (0, 0.24) | 0.053 |  | 0.21 (0.1, 0.32) | 3*10^-4^ |
|  |  |  |  |  |  |  |  |  |  |  |  |  |
| Desire to drink | 0-3 months | 0.11 (-0.04, 0.26) | 0.144 |  | 0.14 (-0.02, 0.31) | 0.086 |  | 0.17 (0.01, 0.34) | 0.043 |  | 0.13 (-0.04, 0.31) | 0.139 |
|  | 3-24 months | 0.02 (-0.14, 0.19) | 0.792 |  | 0.09 (-0.07, 0.26) | 0.274 |  | 0.01 (-0.15, 0.18) | 0.865 |  | 0.06 (-0.11, 0.22) | 0.498 |
|  | 24 months to childhood | 0.02 (-0.13, 0.17) | 0.752 |  | 0.09 (-0.08, 0.25) | 0.310 |  | 0.05 (-0.08, 0.17) | 0.474 |  | 0.02 (-0.1, 0.13) | 0.772 |

Abbreviations: BMI, body mass index.

^a^ Models were adjusted for sex, age at CEBQ, and birth measurements (weight, length/height, BMI, or body fat percentage).

^b^ The β coefficients are derived individual-level growth velocities (change in z-score per month).

**Supplementary Table 3. Associations between the food-avoidant appetitive traits and growth velocities^a^ in 149 children**

| Food-avoidant appetitive traits | Growth periods | Change in weight z-score | |  | Change in length/height  z-score | |  | Change in BMI z-score | |  | Change in body fat percentage z-score | |
| --- | --- | --- | --- | --- | --- | --- | --- | --- | --- | --- | --- | --- |
|  |  | β^b^ (95% CI) | *P* value |  | β^b^ (95% CI) | *P* value |  | β^b^ (95% CI) | *P* value |  | β^b^ (95% CI) | *P* value |
| Satiety responsiveness | 0-3 months | -0.24 (-0.39, -0.09) | 0.002 |  | -0.06 (-0.22, 0.11) | 0.486 |  | -0.22 (-0.39, -0.05) | 0.013 |  | -0.24 (-0.43, -0.06) | 0.012 |
|  | 3-24 months | -0.25 (-0.41, -0.09) | 0.002 |  | -0.24 (-0.41, -0.08) | 0.004 |  | -0.2 (-0.37, -0.03) | 0.019 |  | -0.18 (-0.35, 0) | 0.054 |
|  | 24 months to childhood | -0.34 (-0.49, -0.2) | 8*10^-6^ |  | -0.27 (-0.44, -0.11) | 0.002 |  | -0.25 (-0.38, -0.12) | 2*10^-4^ |  | -0.2 (-0.32, -0.08) | 0.002 |
|  |  |  |  |  |  |  |  |  |  |  |  |  |
| Slowness in eating | 0-3 months | -0.06 (-0.24, 0.11) | 0.465 |  | 0.02 (-0.17, 0.2) | 0.862 |  | -0.06 (-0.26, 0.13) | 0.517 |  | -0.11 (-0.32, 0.1) | 0.315 |
|  | 3-24 months | -0.02 (-0.21, 0.16) | 0.808 |  | -0.08 (-0.27, 0.11) | 0.397 |  | 0.03 (-0.16, 0.22) | 0.785 |  | 0 (-0.2, 0.2) | 0.997 |
|  | 24 months to childhood | -0.16 (-0.33, 0.02) | 0.075 |  | -0.11 (-0.3, 0.08) | 0.256 |  | -0.11 (-0.25, 0.04) | 0.15 |  | -0.1 (-0.24, 0.04) | 0.147 |
|  |  |  |  |  |  |  |  |  |  |  |  |  |
| Emotional undereating | 0-3 months | 0.1 (-0.07, 0.27) | 0.25 |  | 0.15 (-0.03, 0.33) | 0.097 |  | -0.07 (-0.26, 0.12) | 0.49 |  | -0.04 (-0.24, 0.17) | 0.732 |
|  | 3-24 months | 0.08 (-0.1, 0.26) | 0.391 |  | 0.13 (-0.05, 0.32) | 0.162 |  | -0.04 (-0.23, 0.15) | 0.65 |  | 0.01 (-0.19, 0.2) | 0.935 |
|  | 24 months to childhood | 0.07 (-0.09, 0.24) | 0.396 |  | 0.04 (-0.14, 0.22) | 0.68 |  | 0.02 (-0.13, 0.16) | 0.831 |  | 0.03 (-0.11, 0.16) | 0.689 |
|  |  |  |  |  |  |  |  |  |  |  |  |  |
| Food fussiness | 0-3 months | -0.07 (-0.28, 0.14) | 0.513 |  | -0.03 (-0.25, 0.2) | 0.823 |  | 0.01 (-0.23, 0.25) | 0.947 |  | 0.04 (-0.22, 0.29) | 0.788 |
|  | 3-24 months | 0 (-0.23, 0.22) | 0.968 |  | -0.05 (-0.28, 0.18) | 0.675 |  | 0 (-0.23, 0.24) | 0.969 |  | 0.04 (-0.2, 0.28) | 0.747 |
|  | 24 months to childhood | -0.13 (-0.33, 0.08) | 0.219 |  | -0.19 (-0.42, 0.04) | 0.113 |  | -0.03 (-0.21, 0.15) | 0.739 |  | -0.03 (-0.2, 0.14) | 0.735 |

Abbreviations: BMI, body mass index.

^a^ Models were adjusted for sex, age at CEBQ, and birth measurements (weight, length/height, BMI, or body fat percentage).

^b^ The β coefficients are derived individual-level growth velocities (change in z-score per month).
